# Supplementary material for: Initial Experience of Atezolizumab Plus Bevacizumab for Unresectable Hepatocellular Carcinoma in Real-World Clinical Practice
Source: Cancers (Basel). 2021 Jun 3;13(11):2786. doi: 10.3390/cancers13112786 (PMC8199943; doi:10.3390/cancers13112786)
Supplement: Supplementary file 1 [file cancers-13-02786-s001.zip › cancers-1215957-supplementary.pdf]

Supplementary Figure 1

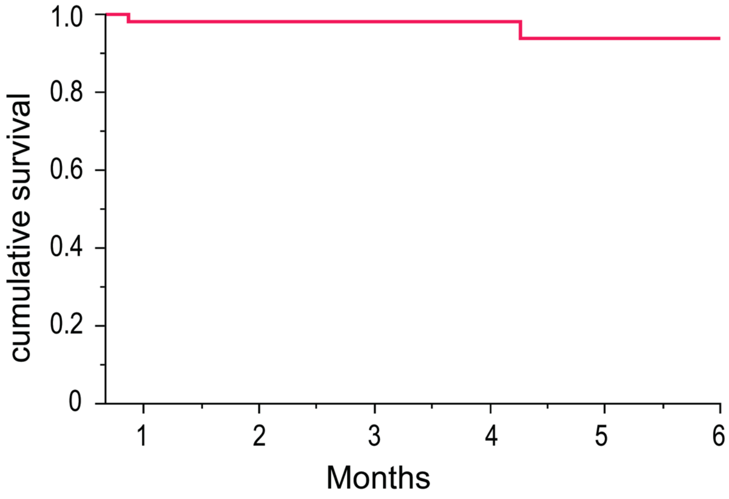

Figure S1. Overall survival of the patients treated with atezolizumab plus bevacizumab in the study

Table S1. Patient characteristics in difference of MTA previous history.

Characteristic	MTA naïve	MTA experienced	*p* value
N	19	32	
Age (years old)	71 (51–80)	71.5 (37–85)	0.69
Sex (female/male)	2/17	4/28	0.83
Etiology (HBV/HCV/nonBnonC)	2/8/9	5/11/16	0.80
Child-Pugh score (5/6/7)	11/6/2	18/12/2	0.82
Child-Pugh grade (A/B)	17/2	30/2	0.58
ALBI score	−2.34 (−2.79– −1.33)	−2.39 (−3.10– −1.49)	0.54
ALBI grade (1/2/3)	3/15/1	8/24	0.28
Tumor size (mm)	41 (11–77)	39 (17–132)	0.47
Up-to-seven criteria (within/beyond)	3/16	3/29	0.78
BCLC stage (B/C)	10/9	14/18	0.53
TNM stage (III/IVA/IVB)	10/5/4	13/2/17	0.029
AFP (ng/mL)	15 (2.1–59,270)	262 (1.3–862,642)	0.24
DCP (mAU/mL)	978 (18–23,088)	2,557 (28–87,529)	0.12

Data are expressed as median (range), or number.

Abbreviations: MTA, molecular targeted agent; ALBI score, Albumin-bilirubin score; BCLC stage, Barcelona Clinic Liver Cancer stage; AFP, α-fetoprotein; DCP, des-γ-carboxy prothrombin

Table S2. Patient characteristics in difference of ALBI grade.

Characteristic	ALBI grade 1	ALBI grade 2 or 3	*p* value
N	11	40	
Age (years old)	70 (48–77)	80 (37–85)	0.63
Sex (female/male)	2/9	4/36	0.47
Etiology (HBV/HCV/nonBnonC)	2/5/4	21/5/14	0.63
Tumor size (mm)	45 (22–83)	40 (11–132)	0.67
Up-to-seven criteria (within/beyond)	1/10	5/35	0.65
BCLC stage (B/C)	5/6	19/21	0.90
TNM stage (III/IVA/IVB)	5/2/4	18/5/17	0.87

AFP (ng/mL)	429 (3.9–59,270)	38 (1.3–862,642)	0.48
DCP (mAU/mL)	3610 (29–52,108)	1114 (18–87,529)	0.70

Data are expressed as median (range), or number.

Abbreviations: ALBI score, Albumin-bilirubin score; BCLC stage, Barcelona Clinic Liver Cancer stage; AFP, α-fetoprotein; DCP, des-γ-carboxy prothrombin.
